# Comparison of the effect of two recruitment manoeuvres to conventional ventilation on lung atelectasis in paediatric laparoscopic surgery- a prospective randomised controlled trial

**DOI:** 10.1186/s12871-024-02596-5

**Published:** 2024-06-22

**Authors:** Aditi Jain, Neerja Bhardwaj, Sandhya Yaddanapudi, Indu Mohini Sen, Preethy Mathew

**Affiliations:** 1 Department of Anesthesia and Intensive Care, Government Medical College and Hospital, Chandigarh, India; 2 Department of Anesthesia and Intensive Care, Postgraduate Institute of Medical Education and Research, Chandigarh, India

**Keywords:** Paediatric minimally invasive, Ventilation strategy, Recruitment

## Abstract

**Background:**

There is a high incidence of pulmonary atelectasis during paediatric laparoscopic surgeries. The authors hypothesised that utilising a recruitment manoeuvre or using continuous positive airway pressure may prevent atelectasis compared to conventional ventilation.

**Objective:**

The primary objective was to compare the degree of lung atelectasis diagnosed by lung ultrasound (LUS) using three different ventilation techniques in children undergoing laparoscopic surgeries.

**Design:**

Randomised, prospective three-arm trial.

**Setting:**

Single institute, tertiary care, teaching hospital.

**Patients:**

Children of ASA PS 1 and 2 up to the age of 10 years undergoing laparoscopic surgery with pneumoperitoneum lasting for more than 30 min.

**Intervention:**

Random allocation to one of the three study groups:

*CG group:* Inspiratory pressure adjusted to achieve a TV of 5–8 ml/kg, PEEP of 5 cm H_2_O, respiratory rate adjusted to maintain end-tidal carbon dioxide (ETCO_2_) between 30-40 mm Hg with manual ventilation and no PEEP at induction.

*RM group:* A recruitment manoeuvre of providing a constant pressure of 30 cm H_2_O for ten seconds following intubation was applied. A PEEP of 10 cm H_2_O was maintained intraoperatively.

*CPAP group:* Intraoperative maintenance with PEEP 10 cm H_2_O with CPAP of 10 cm H_2_O at induction using mechanical ventilation was done.

**Outcome measures:**

Lung atelectasis score at closure assessed by LUS.

**Results:**

Post induction, LUS was comparable in all three groups. At the time of closure, the LUS for the RM group (8.6 ± 4.9) and the CPAP group (8.8 ± 6.8) were significantly lower (*p* < 0.05) than the CG group (13.3 ± 3.8). In CG and CPAP groups, the score at closure was significantly higher than post-induction. The PaO_2_/FiO_2_ ratio was significantly higher (*p* < 0.05) for the RM group (437.1 ± 44.9) and CPAP group (421.6 ± 57.5) than the CG group (361.3 ± 59.4) at the time of pneumoperitoneum.

**Conclusion:**

Application of a recruitment manoeuvre post-intubation or CPAP during induction and maintenance with a high PEEP leads to less atelectasis than conventional ventilation during laparoscopic surgery in paediatric patients.

Trial registry.

CTRI/2019/08/02058.

## Introduction

General anaesthesia (GA) has been shown to have a detrimental effect on pulmonary mechanics, leading to a loss of functional residual capacity (FRC), thereby inducing pulmonary atelectasis. In the paediatric population, the effect of GA on FRC is aggravated due to their compliant chest wall and easily collapsible airways [1].

The creation of pneumoperitoneum in laparoscopic surgeries leads to a cephalad displacement of the diaphragm, which is thought to further increase shunt fraction and dead space [2]. Lung-protective ventilation strategy (LPVS) is used in laparoscopic surgeries, where administration of lower tidal volume (TV), use of sustained positive end-expiratory pressure (PEEP), and the decreased concentration of inhaled oxygen (FiO_2_) is practised to avoid barotrauma or volutrauma to lungs caused by the increased abdominal pressures [3]. However, LPVS leads to a steady decline in FRC and the development of alveolar collapse [4]. This atelectasis and loss of alveolar volume leads to an increase in postoperative complications, and hence efforts should be made to minimise atelectasis during the perioperative period [5].

The incidence of pulmonary atelectasis in infants has been reported to exceed 50% within the first minute of induction of GA, and the incidence increases further during laparoscopic surgeries [6]. Application of a recruitment manoeuvre can help decrease atelectasis in the dependent portions of the lungs. Another possible approach to combat atelectasis can be by using a sustained higher continuous positive pressure to prevent collapse during periods of high oxygen delivery during spontaneous/assisted ventilation such as induction and extubation and during the period of capnoperitoneum [7, 8]. However, no study in the paediatric population has compared these two methods of lung recruitment.

Ultrasonographic (USG) imaging has recently emerged as a reliable modality for assessing the regions of atelectasis in the lungs. It offers a point of care technology, is non-invasive and does not pose any radiation hazard to the patient. USG has been validated as having sufficient sensitivity to detect anaesthesia-induced atelectasis compared to Magnetic Resonance Imaging (MRI) in the paediatric population [9].

This study was planned to analyse the extent of development of atelectasis in children undergoing laparoscopic surgeries under general anaesthesia and to compare the effect of the application of a recruitment manoeuvre and different levels of CPAP/PEEP during induction and maintenance on the development of atelectasis by comparing the lung ultrasound score at the end of surgery.

## Material and methods

### Aim and objectives

The aim of this study was to assess the effect of different ventilation strategies in paediatric laparoscopies on lung parameter. The primary objective was the comparison of lung ultrasound score at end of surgery and secondary objectives included comparison of ventilatory, blood gas and haemodynamic parameter.

#### Study design and participants

We performed a prospective randomised controlled trial over 24 months in 51 children. 

#### Ethics approval and consent to participate

Institutional Ethics Committee (Intramural) of Postgraduate Institute of Medical Education and Research, Chandigarh, India, approved the study vide IEC/2019/001369 on 28.06.2019.Consent for participation and publication was taken from the guardians/parents.The protocol was registered prospectively in the Clinical Trial Registry of India (CTRI/2019/08/020583). The first subject was recruited on 06/08/2019.

Children of ASA PS 1 and 2 up to the age of 10 years undergoing laparoscopic surgery with pneumoperitoneum lasting for more than 30 min under general endotracheal anaesthesia were included in this study. Children with a recent history of upper respiratory tract infection (defined as symptoms present in the last two weeks), family history of malignant hyperthermia or those whose surgeries required positions other than supine were excluded from this study. Written informed consent was obtained from the child’s parent or legal guardian.

#### Randomisation and allocation

Children were randomly allocated to one of the three study groups using a computer-generated random number table: Conventional group (CG group), Recruitment manoeuvre group (RM group), and continuous positive airway pressure group (CPAP group). Concealment of random allocation was done using opaque envelopes. The ventilatory protocols in the three groups were as follows:

- *CG group*: No CPAP was used at induction and PEEP of 5 cm H_2_O was applied after tracheal intubation. Intraoperative ventilation was with inspiratory pressure (PIP) to achieve a tidal volume of 5–8 ml/kg,, and respiratory rate (RR) adjusted to maintain end-tidal carbon dioxide (ETCO_2_) between 30-40 mm Hg.
- *RM group*: No CPAP was used at induction, a recruitment manoeuvre was applied following intubation with a constant pressure of 30 cm H_2_O for ten seconds and PEEP of 10 cm H_2_O was maintained intraoperatively. The rest of the ventilation parameters were similar to the conventional group.
- *CPAP group*: CPAP of 10 cm H_2_O was used at induction after the first lung ultrasound and PEEP 10 cm H_2_O was used intraoperatively. using mechanical ventilation. The rest of the ventilation parameters were similar to the conventional group.

The higher PEEP of 10 was decided based on a previous study done by the authors which compared two different levels of PEEP in paediatric laparoscopic surgeries [10].

#### Conduct of Anaesthesia

The children were fasted according to the standard NPO guidelines. The children were premedicated with 0.5 mg/kg oral midazolam. In the operating room, standard ASA monitoring was initiated. Anaesthesia was induced with sevoflurane (1–8%) in 100% oxygen in all the children. Tracheal intubation was performed with an appropriate size cuffed endotracheal tube after neuromuscular blockade with atracurium (0.5 mg/kg). Anaesthesia was maintained with oxygen in air (FiO_2_ 0.4–0.6) and isoflurane (MAC > 1.1). Opioids (fentanyl 2 µg/kg or morphine 0.1 mg/kg) were used for analgesia. An arterial cannula was placed, preferentially in the radial artery.

Ondansetron (0.15 mg/kg) was administered to the patients at the end of surgery as a prophylaxis for postoperative nausea and vomiting (PONV). The neuromuscular blockade was reversed at the end of the surgical procedure using neostigmine (0.05 mg/kg) and glycopyrrolate (0.01 mg/kg).

#### Data collection and outcome variables

The baseline data was obtained for every patient in the pre-operative ward. Lung ultrasound and arterial blood gas analysis were done at four-time points: Immediately after induction but before administration of muscle relaxant, after intubation (recruitment in the case of RM group), after 5 min of pneumoperitoneum and before extubation. Haemodynamics and ventilatory parameters were also recorded at these time points.

#### Lung ultrasound

Lung ultrasound was performed by dividing each hemithorax into six parts using three longitudinal lines (parasternal, anterior, and posterior axillary) and two axial lines, one above the diaphragm and another one 1 cm above the nipples [9]. The linear probe (13–6 MHZ P) of the Sonosite M Turbo ultrasound machine was used to score each of the 12 regions as follows: 0 = normal aeration represented by the presence of lung sliding and A-lines, 1 = presence of few B lines, 2 = multiple coalescent B-lines, 3 = complete loss of aeration with presence of consolidation. The lung ultrasound score (LUS) aeration score was calculated by adding the scores obtained in the 12 lung areas, ranging from normal aeration (0 points) to the worst possible aeration (36 points).

**Fig. 1:**
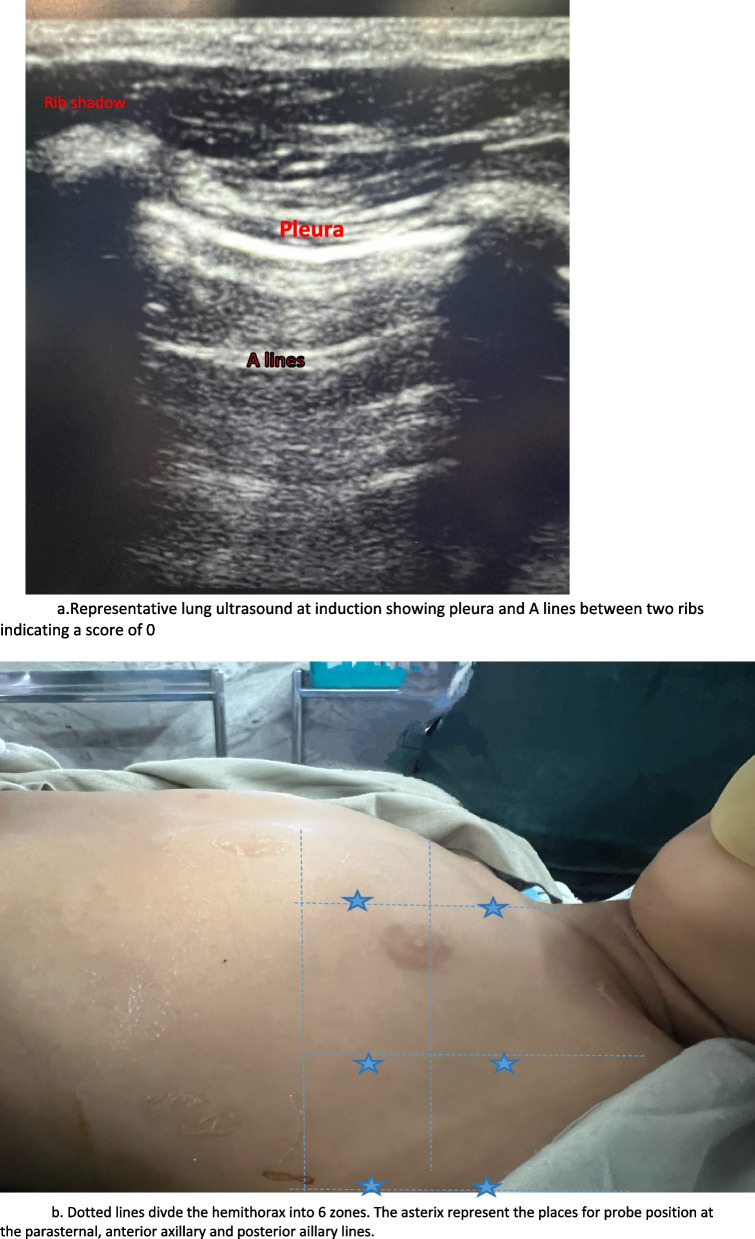
**a** Lung ultrasound with score 0 1b) Position for probe for lung ultrasound in each hemithorax

The primary aim of the study was to compare the atelectasis in the three groups at the end of procedure. The secondary aim was to compare the extent of atelectasis, arterial blood gas parameters, ventilatory parameters, and haemodynamics at the predefined time points within each group and between the three groups. (Fig. 1).

### Statistical analysis

The sample size calculation was based on the study by Acosta et al. in children undergoing laparoscopic surgery who received a recruitment manoeuvre [7] The mean lung USG scores after capnoperitoneum in their study were 8.48 ± 3.22 and 2.52 ± 2.86 in the control and recruitment groups respectively. A sample size of 17 per group was calculated with a two-sided Type 1 error of 0.05 and power of 0.8. It was decided to include 60 patients to adjust for drop outs.Per-protocol analysis was carried out.

Kolmogorov–Smirnov test was used to check for normality.Chi-square test was used for descriptive data for inter group analysis. A two-way ANOVA was carried out to assess the interaction of the ventilation strategies and the time of assessment. Non-parametric data was compared using the Kruskal Wallis test. Post-hoc analysis was done using the Tukey test (HSD). Data at different time points within each group were compared using repeated measure ANOVA. A multilevel mixed linear regression was carried out.

## Results

**Fig. 2:**
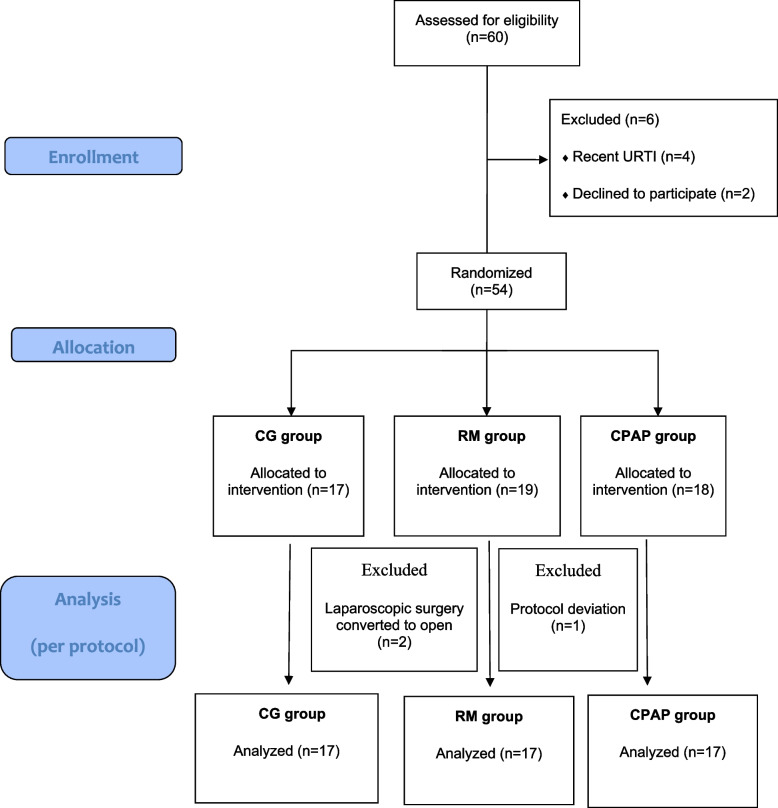
CONSORT diagram

From 25.08.2020 to 10.10. 2021, 60 children undergoing laparoscopic surgery were assessed for eligibility one day prior to surgery. Six children were excluded due to recent URI (*n* = 4), and parental refusal (*n* = 2). Fifty-four children were eventually randomised using a computer generated random number table. The randomisation was concealed in opaque sealed envelopes. The data of 51 children was analysed as per-protocol analysis (Fig. 2). The baseline characteristics of the children in the three groups were comparable, including their age, weight, gender, and type of surgical procedure. The type of surgical procedures performed and the duration of anaesthesia and pneumoperitoneum were similar across the three groups (Table 1).

**Table 1:** Demographic and procedural characteristics

**Variable**		**CG group (*n* = 17)**	**RM group (*n* = 17)**	**CPAP group (*n* = 17)**	***P* value**
Gender (n)*	Male	13	11	12	0.752
	Female	4	6	5	
Weight (kg)		16.7 ± 4.4	20.2 ± 6.6	20.4 ± 7.2	0.151
Age (years)		4.7 ± 2.2	6.3 ± 2.7	6.1 ± 3.2	0.197
Type of surgery (n)*	Pyeloplasty	5	5	2	0.363
	Appendectomy	2	1	5	
	Orchidopexy	2	2	1	
	Cholecystectomy	1	4	3	
	Other renal targeted surgeries	2	4	4	
	Others	5	1	2	
Duration of anaesthesia (min)		176.5 ± 48.6	173.8 ± 68.5	160.5 ± 40.1	0.652
Duration of PnP (min)		126 ± 44.7	120 ± 64.5	106.5 ± 40.6	0.521

Weight, age and duration of anaesthesia and pneumoperitoneum (Pnp) expressed as mean ± *SD* Tests used-ANOVA one-way or Chi square test*

### Primary outcome

#### Lung ultrasound score

The LUS were comparable in the three groups during induction of anaesthesia. The lung ultrasound scores at completion of the surgery were significantly lower in the RM group (8.6 ± 4.9) as well as the CPAP group (8.8 ± 6.8) than those in the CG group (13.3 ± 3.8) (*p* = 0.020).There was no significant difference between the LUS scores of the RM and CPAP group. After intubation in the RM group, a recruitment manoeuvre was provided, and the LUS was calculated post manoeuvre. The LUS in the RM group post manoeuvre (3.8 ± 3.9) was significantly less (*p* = 0.033) than the post-intubation score in the CG group (7.9 ± 5.3).

**Fig. 3:**
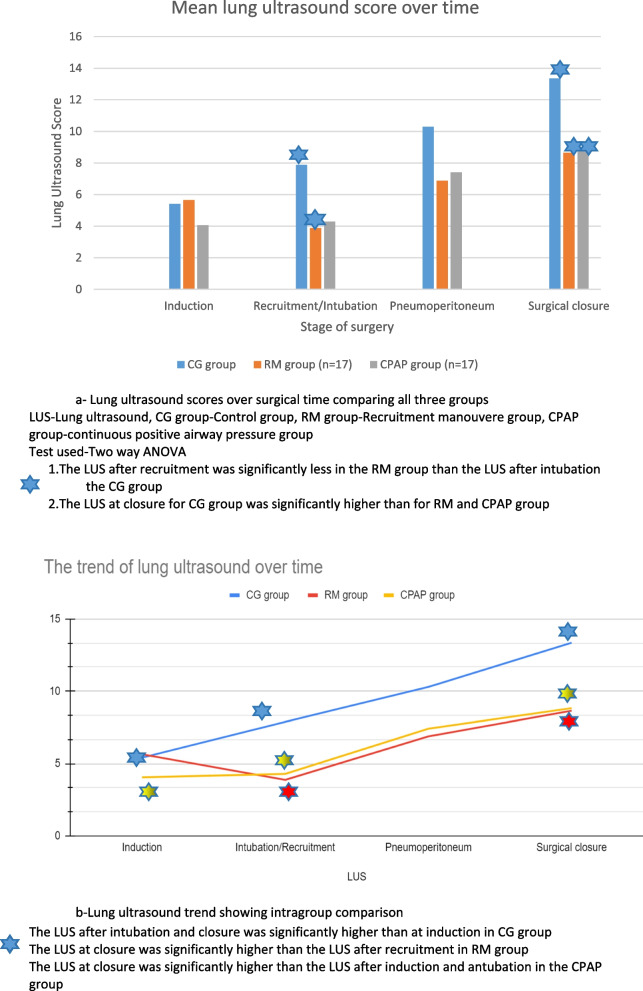
**a** Integroup comparison of lung ultrasound scores **b**) Trend of lung ultrasound score in each group

In the CG group, the score at closure (13.3 ± 3.8) and post creation of pneumoperitoneum (10.3 ± 5) was significantly higher (*p* =  < 0.05) than that at the time of induction (5.4 ± 5.4). In the RM group post recruitment, there was a decrease in the score compared to induction (3.8 ± 3.9). However, it increased from baseline by the time of surgical closure (8.6 ± 4.9).(Fig. 3).

On applying the two-way ANOVA, we found that while both the factors of providing different ventilatory strategies and the surgical steps influenced the LUS, they were not interrelated.A multilevel mixed linear regression analysis deduced that the lung ultrasound score was most significantly affected by the surgical steps and time duration (*p* < 0.001). The difference in the groups was significant(*p* = 0.047).

### Secondary outcomes

#### Arterial blood gas analysis

Post induction and intubation, there was no specific difference between the PaO2/FiO2 ratio of the three groups. At the time of pneumoperitoneum, the PaO2/FiO2 ratio was significantly higher for the RM group (437.1 ± 44.9) and CPAP group (421.6 ± 57.5) than the CG group (361.3 ± 59.4).The difference was also found to be of significance across the three groups (*p* = 0.02) and at different time points (*p* < 0.001) when the multilevel mixed linear regression analysis was done No significant difference was found between the EtCO2, PaCO2 or EtCO2-PaCO2 gradient between the three groups (Table 3).

#### Ventilatory parameters

The driving pressure at the time of pneumoperitoneum and closure needed to generate adequate tidal volume in the RM group (10.8±1.3, 8.3±1.5) was significantly lower than that in the CG group(13.6±2.5, 11.3±3.7). The compliance measured via spirometer at the time of closure as well as pneumoperitoneum was higher in both the RM(17.9 ± 7.1, 13.2 ± 4.3) and CPAP groups (16.8 ± 7.6,13.5 ± 6.0) when compared with the CG group (12.5 ± 6.5, 9.1 ± 3.6). However, this difference was not statistically significant (Table 2). The tidal volume per kg and respiratory rate,across the three groups was comparable after intubation and closure during surgery.(*p* = 0.125,*p* = 0.231).

**Table 2:** Secondary outcomes

	CG group (*n* = 17)	RM group (*n* = 17)	CPAP group (*n* = 17)	*P* value
PaO_2_				
Induction	331 ± 45	351 ± 30	310 ± 42	0.275
Intubation/Recruiment	201 ± 11	182 ± 55	219 ± 61	0.565
Pneumoperitoneum	179 ± 20	158 ± 30	210 ± 23	0.014
Closure	180 ± 24	263 ± 95	202 ± 42	0.074
PaO_2_/FiO_2_ Ratio				
Induction	333 ± 49	339 ± 41	314 ± 51	0.352
Recruitment	385 ± 70	444 ± 74	388 ± 77	0.785
Pneumoperitoneum	376 ± 63	429 ± 44	432 ± 52	0.021
Closure	368 ± 71	425 ± 61	394 ± 72	0.124
Driving pressure (cm H_2_O)				
Intubation	8 ± 1	8 ± 1	8 ± 1	0.763
Trocar insertion	9 ± 2	8 ± 1	9 ± 2	0.460
Pnp	13 ± 2	10 ± 1*	12 ± 1*	0.0007
Closure	11 ± 3	8 ± 1*	9 ± 1	0.0045
Compliance (ml/cm H_2_O)				
Recruitment	NA	16 ± 5	NA	
Trocar insertion	14 ± 5	14 ± 4	15 ± 7	0.977
Pneumoperitoneum	9 ± 3	12 ± 3	13 ± 6	0.318
Closure	14 ± 6	16 ± 6	16 ± 7	0.854
PaCO_2_				
Induction	48 ± 2	51 ± 5	43 ± 11	0.293
Intubation/Recruiment	43 ± 0	44 ± 2	40 ± 16	0.233
Pneumoperitoneum	43 ± 5	48 ± 3	42 ± 4	0.871
Closure	39 ± 8	41 ± 8	42 ± 8	0.410
EtCO_2_				
Induction	34 ± 0	37 ± 1	35 ± 8	0.948
Intubation/Recruitment	36.5 ± 0.7	35 ± 5	36 ± 3	0.208
Pneumoperitoneum	38.3 ± 3.5	38 ± 0	37 ± 3	0.612
Closure	35.7 ± 5.7	35 ± 1	36 ± 2	0.869
PaCO_2_-EtCO_2_				
Induction	11 ± 8	9 ± 8	7 ± 7	0.470
Intubation/Recruiment	6 ± 6	3 ± 3	5 ± 4	0.446
Pneumoperitoneum	5 ± 5	4 ± 6	5 ± 5	0.710
Closure	3 ± 4	3 ± 4	6 ± 6	0.188
Tidal volume(ml/kg)				
Intubation	5 ± 1	5	5	0.812
Pneumoperioneum	6 ± 1	5	6	0.209
Closure	6 ± 0	6	6	0.098
Respiratory rate (per minute)				
Intubation	20 ± 3	20 ± 4	17 ± 4	0.084
Pneumoperitoneum	22 ± 4	19 ± 4	18 ± 4	0.096
Closure	19 ± 4	19 ± 4	21 ± 5	0.236

Data presented as mean ± SD

^*^Statistically less compared to CG groupStatistical Test used- ANOVA

#### Haemodynamic parameters

No recruitment manoeuvre needed to be abandoned due to hypotension or haemodynamic instability. The haemodynamic parameters were comparable across all three groups at various protocol stages (Table 3). While there was an increase in heart rate and blood pressure at intubation and pneumoperitoneum creation, it was within 20% of the baseline and not clinically significant.

**Table 3:** Haemodynamic variables

	**CG group (*n* = 17)**	**RM group (*n* = 17)**	**CPAP group (*n* = 17)**	***P* value**
**Heart Rate (bpm)**				
Baseline	108.0 ± 20.9	107.5 ± 21.5	106.2 ± 18.7	0.971
Induction	110.4 ± 23.2	103.8 ± 17.1	104.4 ± 19.1	0.654
Intubation	120.3 ± 20.7*	118.07 ± 20.1*	116 ± 21.9*	0.872
Trocar insertion	127.9 ± 24.3*	125.3 ± 14.0*	128.5 ± 17.5*	0.890
Pneumoperitoneum	121.7 ± 31.0*	115.5 ± 22.7*	124.7 ± 18.2*	0.595
Closure	105.7 ± 17.5	106.5 ± 19.4	106.3 ± 13.4	0.996
**SBP (mm Hg)**				
Induction	85.4 ± 11.5	86.6 ± 10.3	86.9 ± 11.7	0.924
Intubation	90.6 ± 13.5	96.2 ± 11.6	91.6 ± 13.3	0.407
Trocar insertion	95.1 ± 14.7	101.0 ± 16.3	92.8 ± 11.8	0.235
Pneumoperitoneum	96.5 ± 11.7	104.5 ± 18.0	99.3 ± 16.3	0.325
Closure	94.8 ± 10.1	101.6 ± 14.7	95.8 ± 13.5	0.208
**Diastolic blood pressure (mm Hg)**				
Induction	48.7 ± 10.6	46.3 ± 10.1	54.2 ± 11.1	0.096
Intubation	53 ± 12.0	57.1 ± 12.2	56.4 ± 12.3	0.573
Trocar insertion	58.2 ± 17.2	58.7 ± 16.5	57.8 ± 13.1	0.986
Pneumoperitoneum	62.2 ± 16.0	66.4 ± 19.3	63.7 ± 16.0	0.775
Closure	60.3 ± 9.8	64.8 ± 14.9	61 ± 13.6	0.548

*SBP*, DBP expressed as mean ± SD

Test used -ANOVA

## Discussion

Our study found that providing a recruitment manoeuvre post-intubation or a continuous positive pressure during induction significantly decreased lung atelectasis as quantified by lung ultrasound compared to conventional ventilation in children under ten years old undergoing laparoscopic surgeries. The benefit of recruitment manoeuvre followed by increased PEEP over conventional ventilation is also reflected in the study by Acosta et al. in children undergoing laparoscopic surgeries. They found only 19% of their recruitment group to have significant atelectasis compared to 80% of their control group [7]. However, while Acosta et al. found that their recruitment manoeuvre decreased the atelectasis significantly during and after capnoperitoneum, this was not reflected in our study. This may be attributed to the fact that Acosta et al. did not measure LUS post recruitment as opposed to our study. We believe that the increased PEEP had a significant benefit for the lung aeration as opposed to the recruitment manoeuvre alone.While there was a decrease in loss of aeration post recruitment in our study, it was not absolute. Song et al. also found the consolidation and B-line scores in their recruitment group were significantly lower at surgical closure than the control group [11].

Recruitment manoeuvres and application of increased PEEP are some of the methods to combat lung atelectasis. A recruitment manoeuvre is performed to improve the transpulmonary pressure beyond that offered by tidal volume ventilation. It maximises the number of alveoli participating in the gas exchange to decrease shunt fraction and stabilise lung volume [4]. To be effective, a recruitment manoeuvre must apply sufficient inspiratory pressure to open collapsed areas of the lung, followed by an adequate level of PEEP to keep the alveoli open. The use of PEEP itself has decreased loss of FRC, airway closure, and atelectasis formation. Without PEEP, the lung mechanics shift to the less compliant part of the pressure–volume curve where lung collapse may occur. The optimal PEEP level helps keep the alveoli aerated and thus preserves FRC without compromising the hemodynamics [1].

We found that the lung ultrasound score at surgical wound closure was significantly higher than that post recruitment in the RM group despite being considerably lower than that in the CG group. This indicates that while the recruitment manoeuvre and increased PEEP does not prevent atelectasis altogether, it does contribute to a higher degree of protection from atelectasis than that offered by conventional ventilation. This is also supported by the study of Song et al., who witnessed juxta pleural consolidation in all infants at the end of surgery despite giving them an ultrasound-guided recruitment manoeuvre. While they suggested that a PEEP of 5 may not be sufficient to prevent collapse post recruitment manoeuvre, our study had similar findings despite a fixed higher PEEP [10] Similar results were also reported by Acosta et al., who found that in 4 out of the six children who underwent laparoscopy, a recruitment manoeuvre and a subsequent sustained higher PEEP failed to obliterate atelectasis [7]. We hypothesise that possibly an individualised titrated PEEP, as opposed to a standard PEEP post recruitment, may be more successful in preventing atelectasis.

In our study, the lung ultrasound scores of the CPAP group were comparable to the RM group at all time points. This indicates that a continuous positive pressure of 10 cm H_2_O during induction is beneficial in countering the loss of aeration caused by the pneumoperitoneum. However, there was still a significant increase in lung atelectasis during pneumoperitoneum and during closure than that during induction.

We chose to measure arterial blood gases for a more accurate representation of the oxygenation and carbon dioxide present in the blood. Repeat measurements helped provide a trend of the same over the course of laparoscopy. As far as the authors know, this is the first study to document this trend in laparoscopic paediatric surgeries. The PaO_2_/FiO_2_ratio post development of pneumoperitoneum was significantly less in the CG group than in the RM and CPAP group. This indicates the development of more shunts and dead space in the CG group instead of the RM and CPAP group. Although all the ratios were more than 300, indicating optimum ventilation and perfusion, this difference might become clinically significant in children with coexisting pulmonary disease.

The driving pressure of the CG group was significantly more than that of either the RM or CPAP group during pneumoperitoneum. This was probably secondary to the fact that the RM and CPAP group had a higher PEEP than the CG group, resulting in already distended alveoli and the requirement of lower pressures for ventilation. On the other hand, the driving pressures required after induction and post-intubation across all three groups were similar.

No child in the recruitment group suffered significant hypotension, and the recruitment could be completed in all children successfully. Hence, the recruitment manoeuvre and a PEEP of 10 were tolerated well by all children without compromising their hemodynamics. In previous studies, too, recruitment manoeuvres did not lead to significant hypotension, deeming them safe to be used in ASA 1 and 2 patients. In the study by Acosta et al., they did not find any hemodynamic compromise in any of their cases [7]. Similarly, none of the infants in the study by Song et al. showed any hemodynamic instability [10]. In a study done in 32 ventilated paediatric patients in the critical care unit; Duff et al. reported transient bradycardia in 2/93 recruitment performed and increased intracranial compliance in three subjects [12]. Although they concluded that manoeuvres are safe and beneficial in critically ill children, these side effects should be considered, and use of manoeuvres and elevated PEEP should be judiciously administered in the setting of ASA 3 and 4 patients. Large scale population studies in children with comorbidities can better establish the safety and efficacy of these recruitment strategies under general anaesthesia.

### Strengths and limitations

In our study, the same anaesthetist performed lung ultrasound in all the patients to exclude inter-observer variability. We used an established lung ultrasound score to quantify atelectasis objectively. Repeated scans were done at different times during the surgery that helped establish a more apparent trend to understand the components leading to atelectasis. By performing concurrent blood gas analysis, objectivity was added to the study.

Our study has a few limitations. We did not obtain a baseline LUS before induction and assumed that all the children recruited in the study had normal lung aeration. An awake child is unlikely to be cooperative in the preoperative period without premedication. Hence, we did not obtain a preoperative image. Also, hyperinflation is not detected by lung ultrasound. We limited the peak inspiratory pressure to avoid the same. The assessor was not blinded to the ventilatory strategy as the ultrasound images were obtained and assessed in real-time during the surgery. Fluid balance in critically ill patients has been seen to affect the lung ultrasound, although its effect on surgical paediatric population is not well studied.We did not study the correlation of fluid balance with lung ultrasound scores.

Our study can pave the way for future research. A larger-scale study may help establish the superiority of one ventilation strategy over the other. Titration of PEEP to detect optimum PEEP per patient may provide a more effective method for preventing atelectasis per patient. Ultrasound-guided recruitment strategies may also be investigated to decrease atelectasis that has set in during induction. The efficacy and safety of these ventilation strategies need to be tested in ASA 3 and 4 patients.

## Conclusion

Our study indicated that applying a recruitment manoeuvre post-intubation or CPAP during induction with 100% oxygen and following them up with a high PEEP leads to less atelectasis than conventional ventilation during laparoscopic surgery. These strategies also lead to better lung compliance and oxygenation during pneumoperitoneum without hemodynamic compromise in the paediatric population.

## Data Availability

The datasets used and/or analysed during the current study is available from the corresponding author on reasonable request.
